# A phase II clinical trial of the Aurora and angiogenic kinase inhibitor ENMD-2076 for previously treated, advanced, or metastatic triple-negative breast cancer

**DOI:** 10.1186/s13058-018-1014-y

**Published:** 2018-08-02

**Authors:** Jennifer R. Diamond, S. G. Eckhardt, Todd M. Pitts, Adrie van Bokhoven, Dara Aisner, Daniel L. Gustafson, Anna Capasso, Sharon Sams, Peter Kabos, Kathryn Zolman, Tiffany Colvin, Anthony D. Elias, Anna M. Storniolo, Bryan P. Schneider, Dexiang Gao, John J. Tentler, Virginia F. Borges, Kathy D. Miller

**Affiliations:** 1 University of Colorado Cancer Center, Aurora, CO USA; 2 Department of Oncology, University of Texas at Austin, Dell Medical School, Austin, TX USA; 3 Indiana University Melvin and Bren Simon Cancer Center, Indianapolis, IN USA; 4 Division of Medical Oncology, University of Colorado Anschutz Medical Campus, University of Colorado Cancer Center, 12801 East 17th Avenue, Mailstop 8117, Aurora, CO 80045 USA

**Keywords:** Breast cancer, ENMD-2076, Aurora kinase inhibitor, Triple negative

## Abstract

**Background:**

Triple-negative breast cancer (TNBC) remains an aggressive breast cancer subtype with limited treatment options. ENMD-2076 is a small-molecule inhibitor of Aurora and angiogenic kinases with proapoptotic and antiproliferative activity in preclinical models of TNBC.

**Methods:**

This dual-institution, single-arm, two-stage, phase II clinical trial enrolled patients with locally advanced or metastatic TNBC previously treated with one to three prior lines of chemotherapy in the advanced setting. Patients were treated with ENMD-2076 250 mg orally once daily with continuous dosing in 4-week cycles until disease progression or unacceptable toxicity occurred. The primary endpoint was 6-month clinical benefit rate (CBR), and secondary endpoints included progression-free survival, pharmacokinetic profile, safety, and biologic correlates in archival and fresh serial tumor biopsies in a subset of patients.

**Results:**

Forty-one patients were enrolled. The 6-month CBR was 16.7% (95% CI, 6–32.8%) and included two partial responses. The 4-month CBR was 27.8% (95% CI, 14–45.2%), and the average duration of benefit was 6.5 cycles. Common adverse events included hypertension, fatigue, diarrhea, and nausea. Treatment with ENMD-2076 resulted in a decrease in cellular proliferation and microvessel density and an increase in p53 and p73 expression, consistent with preclinical observations.

**Conclusions:**

Single-agent ENMD-2076 treatment resulted in partial response or clinical benefit lasting more than 6 months in 16.7% of patients with pretreated, advanced, or metastatic TNBC. These results support the development of predictive biomarkers using archival and fresh tumor tissue, as well as consideration of mechanism-based combination strategies.

**Trial registration:**

ClinicalTrials.gov, NCT01639248. Registered on July 12, 2012.

## Background

Triple-negative breast cancer (TNBC) is an aggressive breast cancer subtype defined by a lack of expression of the estrogen receptor (ER), progesterone receptor, and human epidermal growth factor receptor 2 (HER2) [1]. TNBC accounts for 10–15% of all newly diagnosed breast cancer and is associated with an increased risk of distant metastasis and death compared with other breast cancer subtypes [1–3]. The median duration of first-line therapy for metastatic TNBC is approximately 12 weeks, and the median survival for patients with metastatic disease is 10–13 months [2, 4]. Despite the characterization of biologic subtypes within TNBC, this disease remains critically in need of effective targeted systemic therapies [5].

ENMD-2076 is an orally bioavailable small-molecule inhibitor of angiogenic and mitotic kinases. The antiangiogenic activity of ENMD-2076 is mediated through the inhibition of vascular endothelial growth factor receptors (VEGFRs) and fibroblast growth factor receptors (FGFRs), whereas antiproliferative activity occurs via inhibition of mitotic kinases, including Aurora kinase A (Aur A) [6]. ENMD-2076 is active against preclinical TNBC models, including p53-mutated cancer cell lines and patient-derived tumor xenograft (PDX) models [7, 8]. The purpose of this study was to evaluate the anticancer activity of ENMD-2076 in patients with previously treated locally advanced or metastatic TNBC, to further characterize the side effect profile, and to explore pharmacodynamic changes in serial tumor biopsies.

## Methods

### Study design

This phase II clinical trial was a dual-institution, single-arm, Simon two-stage study of single-agent ENMD-2076 administered orally once daily with continuous dosing (ClinicalTrials.gov identifier NCT01639248). The primary objective of the study was to determine the 6-month clinical benefit rate (CBR), defined as patients with complete response, partial response (PR), or stable disease (SD) lasting for ≥ 24 weeks based on Response Evaluation Criteria in Solid Tumors (RECIST version 1.1) [9]. Secondary objectives included progression-free survival (PFS), objective response rate, safety, and pharmacokinetics (PK). Exploratory objectives included evaluation of p53 mutation status on archival tumor tissue and pharmacodynamic effects of ENMD-2076 in serial tumor tissue samples obtained in a subset of patients. This protocol was approved by the institutional review boards of both institutions, and informed consent was obtained from all patients prior to performing study-related procedures in accordance with federal and institutional guidelines.

### Eligibility criteria

Eligible patients had locally advanced or metastatic TNBC previously treated with one to three prior lines of chemotherapy in the advanced setting. For the purpose of this study, locally advanced breast cancer was defined as unresectable local or regional disease. TNBC was defined as negative for estrogen and progesterone receptor by local pathology report, Allred score ≤ 2, or < 5% weak positive staining. Negative HER2 testing was defined as IHC score 0 or 1+ or fluorescence in situ hybridization ratio < 2.0. Patients were required to have measurable disease by RECIST 1.1 [9], Eastern Cooperative Oncology Group (ECOG) Performance Status of 0–1, and archival tumor tissue available for analysis. Patients also had to be aged 18 years or older and to have adequate hematopoietic, hepatic, and kidney function, defined as hemoglobin ≥ 9 g/dl, absolute neutrophil count ≥ 1500/μl, platelets ≥ 100,000/μl, total bilirubin < 1.5 times the institutional upper limit of normal (ULN), aspartate aminotransferase/alanine aminotransferase and ≤ 2.5 times the ULN or < 5 times the ULN if hepatic metastases were present, and creatinine ≤ 1.5 times the ULN. Brain magnetic resonance imaging (MRI) was required to exclude brain metastasis. For patients undergoing the optional serial tumor biopsies, prothrombin time and activated partial thromboplastin time were required to be within the normal range. Patients were recovered from the expected toxicity of prior treatments and did not require therapeutic doses of any anticoagulant or have any significant cardiac problems.

### Pretreatment evaluation

Prior to the initiation of study treatment, all patients underwent clinical history and physical examination, ECOG Performance Status assessment, vital signs, complete blood count, chemistries, urinalysis, serum pregnancy test (if applicable), coagulation parameters (for patients undergoing tumor biopsies), echocardiogram or multigated acquisition, and baseline tumor assessment with imaging. Archival tissue was submitted for correlative analysis for all enrolled patients. Additionally, serial tumor biopsies were performed for correlative analysis in a subset of patients, with the first occurring before the initiation of study treatment.

### Treatment and dose modifications

Patients were treated with ENMD-2076 250 mg oral once daily with continuous dosing in 4-week cycles until disease progression or unacceptable toxicity occurred. The dose of study drug was selected on the basis of tolerability of ENMD-2076 in a phase II clinical trial in patients with recurrent, platinum-resistant ovarian cancer where the initial ENMD-2076 dose was 325 mg oral once daily for patients with a body surface area (BSA) ≥ 1.65 m^2^ and 275 mg/d for patients with a BSA ≤ 1.64 m^2^ [10]. Due to higher-than-expected rates of treatment-related toxicity resulting in dose delays in that study, the starting dose was reduced to 275 mg/d and 250 mg/d for the two BSA groups, respectively. Taking into account this tolerability data for ENMD-2076 administered on a once-daily continuous dosing schedule, a flat dose of 250 mg/d was selected as the starting dose for this study.

Restaging was performed every two cycles (8 weeks) according to RECIST 1.1. Blood pressure was monitored weekly during the first cycle, every 2 weeks during cycle 2, and every 4 weeks during cycles 3 and beyond. Complete blood count and chemistries were performed every 2 weeks during cycles 1 and 2, then every 4 weeks. Urinalysis was performed every 4 weeks, and if 2+ proteinuria was observed, a spot urine protein/creatinine ratio was calculated and a 24-hour urine sample was collected for quantification of protein. Patients were allowed to continue treatment if the spot urine/protein ratio was ≤ 1 and the 24-hour urine showed ≤ 3.5 g protein/24 h. If the 24-hour urine protein was > 3.5 g, treatment was withheld until a repeat study was ≤ 3.5 g protein/24 h. Thyroid-stimulating hormone was tested on cycle 3, day 1 and subsequently as clinically indicated.

Dosing delays of up to 2 weeks were permitted to allow for recovery from treatment-related toxicities or other intercurrent illness. Longer delays were also allowed in patients experiencing clinical benefit from ENMD-2076 treatment. Two dose reductions were allowed for treatment-related toxicity (dose − 1 was 225 mg/d and dose − 2 was 150 mg/d). The protocol did not dictate specific management strategies for ENMD-2076-related treatment toxicities, including hypertension. The treatment management strategy was at the discretion of the treating physician.

### Pharmacokinetic sampling and assay

Blood samples were collected in sodium heparin tubes prior to the first dose on cycle 1, day 1 and then 4 hours following dosing. Samples were obtained prior to dosing on cycle 2, day 1 and on the day of tumor biopsy. Plasma concentrations of ENMD-2076 and its active metabolite, ENMD-2060, were determined using a validated LC-MS/MS method. The lower limits of quantification were 2.5 ng/ml for ENMD-2076 and 1 ng/ml for ENMD-2060.

### Correlative studies using archival tumor tissue and serial tumor biopsies

Archival tumor tissue was requested from all eligible patients and subject to evaluation for p53 mutation and IHC expression. If multiple samples were available, the most recent tumor tissue was selected for testing unless it was quantitatively insufficient. Microdissection was performed using a stereotactic microscope with hematoxylin-stained slides, and DNA was isolated for sequencing of *TP53* exons 5–8 and 10. Briefly, following microdissection, samples were purified using the QIAamp DSP FFPE Tissue Kit (Qiagen, Valencia, CA, USA). Exons 5–8 and 10 were PCR-amplified followed by Sanger sequencing using the Applied Biosystems BigDye system (Life Technologies, Carlsbad, CA, USA), and capillary electrophoresis was performed on an ABI 3500xL instrument (Applied Biosystems/Thermo Fisher Scientific, Foster City, CA, USA). Sequence analysis was performed using MutationSurveyor software (SoftGenetics, State College, PA, USA). *TP53* alterations were assessed for pathogenicity by first determining whether they represented population polymorphisms using the Exome Aggregation Consortium database (exac.broadinstitute.org). Any alteration with a population minor allele frequency > 1% was excluded from further analysis. All remaining identified alterations were examined for pathogenicity on the basis of classification by the International Agency for Research on Cancer TP53 Database (p53.iarc.fr), and all alterations classified as “deleterious” by this database were considered mutations for the purposes of subsequent analysis.

Serial tumor biopsies were performed in a subset of patients during the first cycle prior to day 1 and at days 14–16. An additional biopsy was obtained at the time of progression in patients responding to treatment. Formalin-fixed, paraffin-embedded samples were analyzed by IHC for Ki-67 (30-9; Ventana Medical Systems, Inc., Tucson, AZ, USA), cleaved caspase 3 (Cell Signaling Technology, Danvers, MA, USA), p53 (Cell Marque, Rocklin, CA, USA), and CD34 (Abcam, Cambridge, MA, USA). Following antigen retrieval and primary antibody incubation, IHC stains were visualized with the UltraView Universal DAB Detection Kit (Ki-67 and p53; Ventana Medical Systems, Inc.) or ImmPRESS HRP Antirabbit IgG (CD34 and cleaved caspase 3; Vector Laboratories, Burlingame, CA, USA). IHC for Ki-67 was scored as follows: low proliferative index ≤ 15% positive cells, intermediate 16–30%, and high > 30%. IHC for p53 was scored as low ≤ 15% positive cells, intermediate 16–30%, and high > 30%. The average number of vessels from three × 20 magnification fields was used as the microvessel density (MVD) score. The cleaved caspase 3 score was determined by evaluating the percentage of stained tumor cells.

Immunofluorescence (IF) was performed in a subset of biopsy samples for 4′,6-diamidino-2-phenylindole (DAPI), p53, p73, and BAX as previously described [8]. In brief, samples were freshly collected, placed into individual cryomolds (Sakura Finetek, Torrance, CA, USA) and embedded in Tissue-Tek optimum cutting compound (Sakura Finetek). Cryomold blocks were frozen in liquid nitrogen and individually cut into 5-μm-thick sections by the University of Colorado Cancer Center Tissue Biobanking and Histology Shared Resource. Slides were fixed in a 1:1 ratio in a solution of methanol and acetone at − 20 °C for 10 minutes, blocked, and incubated with primary antibodies (p53, Cell Signaling Technology; p73, Abcam). Slides were washed three times in 1 × PBS and incubated with secondary antibodies (Alexa Fluor 555 or Alexa Fluor 488; Life Technologies) and counterstained with 300 nM DAPI. Slides were mounted, and images were acquired using the FLUOVIEW FV1000 confocal microscope (Olympus America, Center Valley, PA, USA) at × 60 magnification.

### Statistical methods

The primary endpoint of the study was the 6-month CBR, defined as the sum of patients who experienced complete response, PR, and/or stable disease (SD) for ≥ 24 weeks by RECIST version 1.1. The sample size was determined using a null hypothesis of 10% [11, 12] and an alternate hypothesis of interest to continue single-agent studies in the patient population of 30%. The sample size of 35 yielded a power of 90% to detect this difference with an alpha of 0.05 (one-sided). Patients were considered evaluable for the primary endpoint if they received at least one cycle of therapy and underwent repeat disease assessment. Patients who came off study during the first cycle for toxicity or patient preference without disease progression were not considered evaluable for the primary endpoint. This study used a two-stage study design. The interim analysis (stage 1) was performed after enrollment of 18 patients. If 2 or fewer of the first 18 patients experienced a “success”, then it would be concluded that the treatment does not have sufficient activity for further investigation, and accrual would be terminated. If 3 or more of these 18 patients experienced a “success,” enrollment would continue to 17 additional evaluable patients.

Descriptive statistics were used for PK and toxicity parameters. Fisher’s exact test was used to assess the correlation between p53 mutation and response to treatment.

## Results

### Patients

**Fig. 1:**
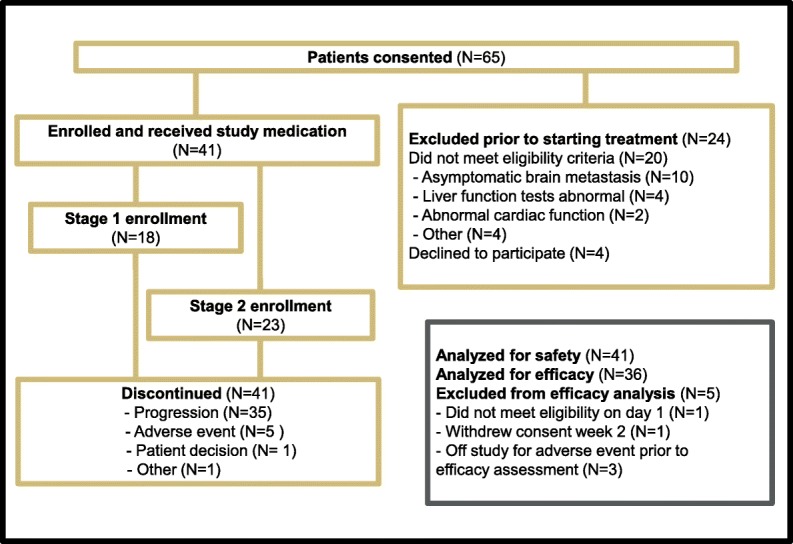
Trial profile and study design

Between July 2012 and October 2016, 65 patients were consented, and 41 patients were enrolled at the University of Colorado Cancer Center (Aurora, CO, USA) and the Indiana University Melvin and Bren Simon Cancer Center (Indianapolis, IN, USA). This included 18 patients enrolled in stage 1 and 23 patients in stage 2 (Fig. 1). A total of 24 patients were consented and excluded prior to starting treatment, most commonly for not meeting the eligibility criteria owing to asymptomatic brain metastasis detected on required screening brain MRI or abnormal liver function tests.

The median age of patients was 54 years (range, 30–73 years); 40 were female, and 1 was male. Patients received, on average, 1.7 prior lines of chemotherapy for locally advanced unresectable or metastatic disease, and 80.5% underwent prior neoadjuvant or adjuvant chemotherapy for localized disease. In addition, 48.8% of patients received prior systemic targeted anticancer therapies, and 85.4% received prior radiation therapy. ECOG Performance Status was 1 in 53.7% of patients and 0 in 46.3% of patients. BRCA1/2 germline mutation status was collected if known; 9.8% had known deleterious mutations, 46.3% were known wild type, and status was unknown in 43.9% of patients. Additional baseline patient characteristics and demographics can be found in Table 1.

**Table 1:** Baseline demographics and patient characteristics

Characteristic	Number of patients (*N* = 41)
Age, years	
Median (range)	54 (30–73)
Sex	
Male	1 (2.4%)
Female	40 (97.6%)
Race	
White	33 (80.5%)
Black or African American	5 (12.2%)
Unknown	3 (7.3%)
Ethnicity	
Hispanic or Latino	6 (14.6%)
Not Hispanic or Latino	30 (73.2%)
Unknown	5 (12.2%)
ECOG Performance Status	
0	19 (46.3%)
1	22 (53.7%)
Prior lines of systemic therapy	
(locally advanced unresectable or metastatic disease) Mean	1.7
1	21 (51.2%)
2	10 (24.4%)
3	10 (24.4%)
Prior neoadjuvant or adjuvant chemotherapy	
Yes	33 (80.5%)
No	8 (19.5%)
Prior targeted systemic anticancer therapy	
Yes	20 (48.8%)
No	21 (51.2%)
Prior radiation therapy	
Yes	35 (85.4%)
No	6 (14.6%)
BRCA1/2 germline deleterious mutation status	
Mutated	4 (9.8%)
Wildtype	19 (46.3%)
Unknown	18 (43.9%)
Number of metastatic sites	
1	16 (39.0%)
2	14 (34.1%)
≥ 3	11 (26.8%)
Sites of metastasis	
Lung	15 (24.4%)
Lymph nodes	20 (48.8%)
Liver	15 (24.4%)
Bone	16 (39.0%)
Chest wall	8 (19.5%)
Other	6 (14.6%)

*ECOG* Eastern Cooperative Oncology Group

### Efficacy

**Fig. 2:**
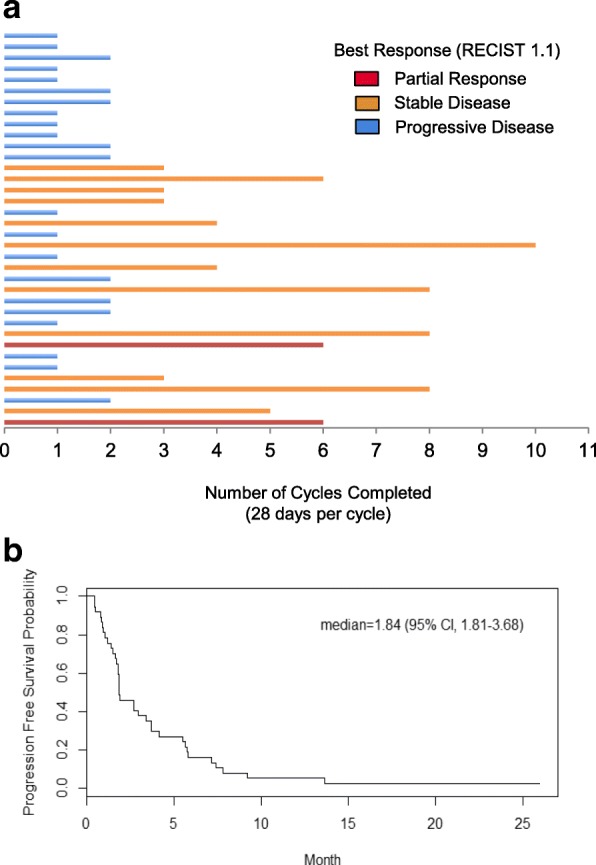
Duration on therapy. **a** Number of cycles of therapy for each patient. Cycles are 28 days. **b** Progression-free survival curve

Thirty-six patients were evaluable per protocol for the primary efficacy analysis. Five patients (12.2%) were not included in the efficacy analysis owing to adverse events leading to discontinuation prior to objective efficacy assessment (*n* = 3), not meeting eligibility criteria on day 1 (*n* = 1), and withdrawal of consent in cycle 1 (*n* = 1) (Fig. 1). The study proceeded to the second stage of enrollment based on observing three 6-month CBR events in stage 1 (*n* = 18 patients). The 6-month CBR in the overall trial was 16.7% (exact 95% CI, 6–32.8%), and the 4-month CBR was 27.8% (exact 95% CI, 14–45.2%) (Table 2). Two patients achieved partial response to treatment, and no complete responses were observed. The average duration of response for patients achieving clinical benefit for 4 months was 6.5 cycles (*n* = 10) (Fig. 2). The median PFS for all patients treated with ENMD-2076 (*n* = 41) was 1.84 months (95% CI, 1.81–3.68) (Fig. 2). The median PFS for all patients evaluable for the efficacy analysis (*n* = 36) was 1.86 months (95% CI, 1.73–3.73).

**Table 2:** Efficacy analysis

Efficacy response	Number of patients (*N* = 36)
Complete response	0 (0%)
Partial response	2 (5.6%)
Stable disease	14 (38.9%)
Progressive disease	20 (55.6%)
4-Month clinical benefit rate (4-CBR)	10 (27.8%)
6-Month clinical benefit rate (6-CBR)	6 (16.7%)

### Safety profile

The most common treatment-related adverse events were hypertension (66%), fatigue (54%), diarrhea (54%), and nausea (49%). Table 3 lists all treatment-related adverse events occurring in 10% or more of patients. Dose reduction occurred in eight patients (20%) for fatigue, hypertension, and proteinuria. The most common grade 3 treatment-related adverse events were hypertension (37.5%) and fatigue (10%). One patient experienced grade 4 hypertension. Five patients (12.2%) discontinued treatment owing to a treatment-related adverse event (Fig. 1).

**Table 3:** Treatment-related adverse events occurring in 10% or more of patients

Adverse event	No. of patients (*N* = 41)	
	All grades	Grade ≥ 3
Hypertension	27 (66%)	16 (39%)
Fatigue	22 (54%)	4 (10%)
Diarrhea	22 (54%)	1 (2%)
Nausea	20 (49%)	0 (0%)
Constipation	10 (24%)	0 (0%)
Headaches	10 (24%)	0 (0%)
Vomiting	9 (22%)	1 (2%)
Mucositis	6 (15%)	0 (0%)
Proteinuria	6 (15%)	3 (7%)
Dysgeusia	5 (12%)	0 (0%)
GERD	5 (12%)	0 (0%)
Anorexia	4 (10%)	0 (0%)

*GERD* Gastroesophageal reflux disease

### Pharmacokinetics

The mean steady-state plasma concentration of ENMD-2076 at cycle 2, day 1 was 441 ± 394 ng/ml. The mean steady-state concentration of its active metabolite, ENMD-2060, was 43.3 ± 10.3 ng/ml. The mean ratio of ENMD-2076 to ENMD-2060 was 10.3 ± 4.9.

Additional PK sampling was performed in patients undergoing serial tumor biopsies on the day of the biopsy. This was performed at cycle 1, days 14–16. PK sampling data was available for ten subjects undergoing serial tumor biopsy, and the mean plasma concentrations of ENMD-2076 and ENMD-2060 were 441 ± 275 ng/ml and 40.1 ± 22.9 ng/ml, respectively.

### Correlative analysis of serial tumor biopsies and archival tumor tissue

A total of 15 patients underwent at least one tumor biopsy analysis. Eight patients had predose and postdose samples (days 14–16) with sufficient tissue for IHC analysis. One patient underwent an additional biopsy at the time of disease progression following treatment for ten cycles.

**Fig. 3:**
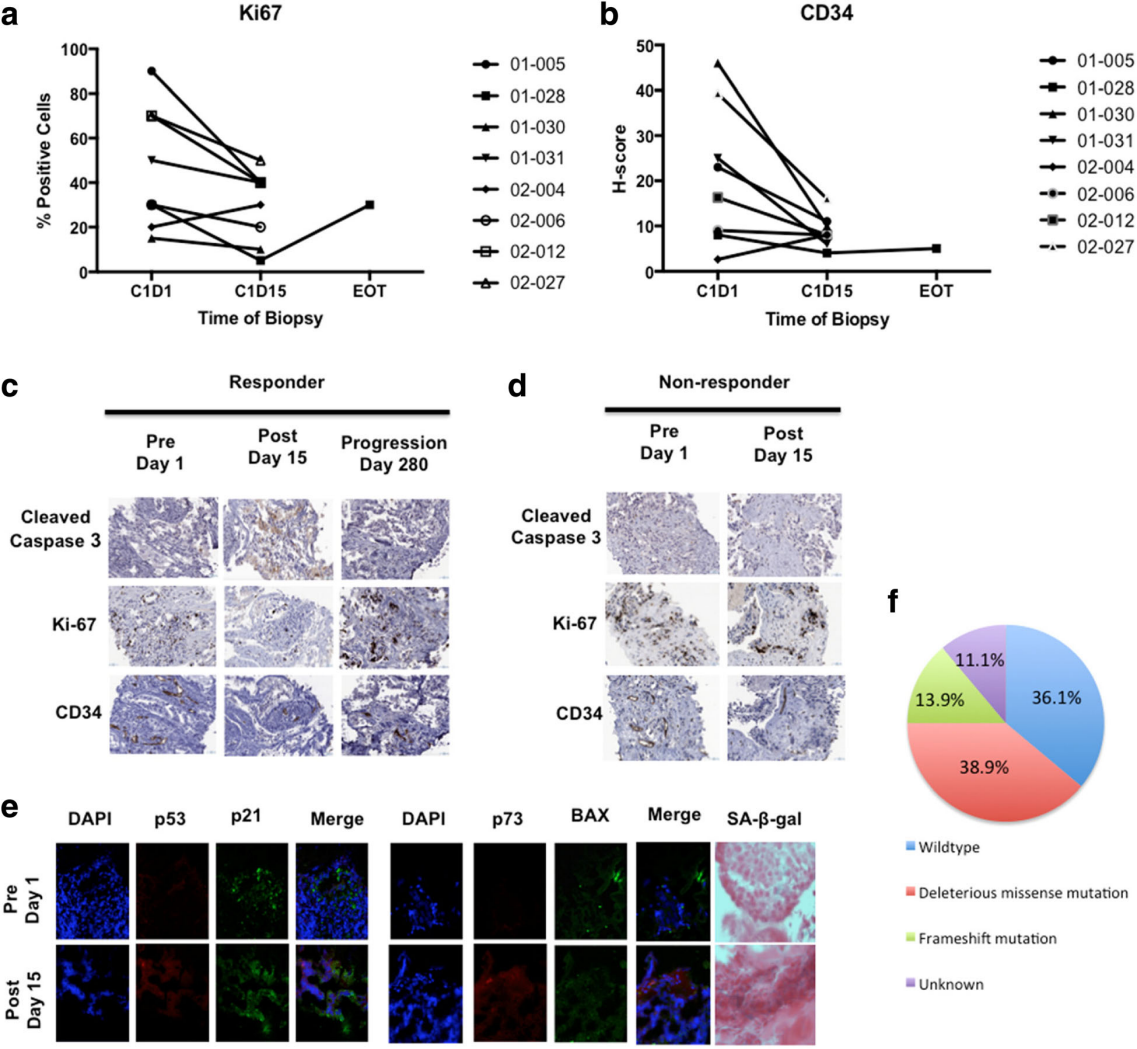
Effects of ENMD-2076 on pharmacodynamic markers in serial tumor biopsies obtained in a subset of patients. **a** Paired samples were available for eight patients at baseline prior to dosing (C1D1) and postdose on days 14–16 (C1D15). An additional sample was obtained in one patient who experienced stable disease for ten cycles followed by progression (end of treatment [EOT]). Tissue was analyzed by IHC for Ki-67 as a marker of cellular proliferation. **b** Nonresponder. Staining was performed as in panel **a**. Note that there is no decrease in proliferation or increase in apoptosis in the nonresponder following ENMD-2076 treatment. Samples were analyzed by IHC for CD34 expression as a marker of microvessel density. Changes were independent of tumor response and clinical benefit to ENMD-2076 treatment. Patients 01-005, 01-030, 02-004, 02-006, 02-012, and 02-027 had progressive disease (PD) at first imaging assessment following two cycles; 01-028 had stable disease (SD) for ten cycles; and 01-031 had SD for four cycles. **c** Immunoflurorescence analysis of tumor biopsies for 4′,6-diamidino-2-phenylindole (DAPI), p53, and p73 in a patient who had stable disease by Response Evaluation Criteria in Solid Tumors (RECIST version 1.1) after two cycles of treatment and then progressed after cycle 3. Patient has a p53 mutation R273S. Note an increase in p53 and p73 following treatment, which is consistent with preclinical findings in patient-derived tumor xenograft models. IHC images from 01-028 responder. Cleaved caspase 3, Ki-67, and CD34 on serial tumor biopsies were used to assess apoptosis, proliferation, and microvessel density, respectively, in a patient responding to ENMD-2076 treatment with prolonged stable disease for ten cycles. Biopsies were obtained prior to treatment, 15 days after treatment, and at the time of disease progression day 280. Formalin-fixed, paraffin-embedded tissue sections were stained with the indicated antibodies, and representative images were obtained at × 20 magnification. Note an increase in cleaved caspase 3 and a decrease in Ki-67 and CD34 in the posttreatment biopsy. At the time of disease progression, these changes were reversed. Changes in (**d**) Ki-67 and (**e**) CD34 (microvessel density) in serial tumor biopsies. Baseline and day 15 samples were available for eight patients. An additional sample was obtained from one patient at the time of progression following prolonged stable disease. Ki-67 and CD34 were assessed using IHC. *SA-β-gal* Senescence-associated β-galactosidase

Analysis of serial tumor biopsies prior to and following 2 weeks of ENMD-2076 (*n* = 8 patients) demonstrated a treatment-induced decrease in cellular proliferation (Ki-67) (Fig. 3a) and MVD (CD34) (Fig. 3b) as assessed by IHC. This was a trend observed in patients regardless of tumor response to treatment by RECIST 1.1. An increase in cleaved caspase 3 as a marker of apoptosis was only observed in association with response to treatment and not in nonresponders (Figs. 3c and d). The posttreatment decrease in Ki-67 and increase in cleaved caspase 3 observed in patient 01-028 (Fig. 3c, responder) was lost at the time of progression at day 280 of treatment, consistent with preclinical modeling of ENMD-2076 activity in TNBC PDX models [8]. An increase in MVD was not observed at the time of acquired progression in this patient’s tumor sample.

On the basis of emerging preclinical data, the final two patients undergoing serial tumor biopsies had their samples processed for IF to investigate changes in p53, p21, p73, BAX, and senescence-associated β-galactosidase (SA-β-gal) following treatment [8]. One patient had sufficient tissue obtained from the pretreatment and posttreatment samples for IF and SA-β-gal. In this patient, an increase in p53 and p73 expression was observed (Fig. 3e), in combination with an increase in SA-β-gal staining. DNA mutational testing of p53 revealed the presence of an R273S mutation in this patient’s tumor, and the patient had SD for three cycles on study followed by progression. An increase in p53 family member expression following treatment is consistent with changes observed in preclinical TNBC PDX models treated with ENMD-2076 and other Aurora kinase inhibitors [8, 13].

Archival tumor tissue was obtained from all enrolled patients, and p53 mutation testing revealed deleterious missense mutations in 38.9%, frameshift mutations in 13.9%, wild type in 36.1%, and status unknown for 11.1% (Fig. 3f). Of the six patients who had PR or SD for > 6 months, 83% had a deleterious mutation in p53, as compared with 46% of those who did not meet that efficacy endpoint (*p* = 0.18). Archival tumor tissue samples were also subjected to p53 IHC. High p53 expression was defined as > 30% of cells expressing p53. High p53 expression was present in 16 (50%) patients who had tissue available for analysis (*n* = 32), and high p53 expression plus p53 mutation was present in 11 of 32 (34.4%). High p53 expression was observed in 50% of patients meeting the 6-month CBR endpoint and those not meeting this efficacy endpoint, showing no correlation between high expression of p53 by IHC and efficacy.

## Discussion

This study demonstrates that ENMD-2076 has durable clinical activity in a subset of patients with previously treated metastatic TNBC. We observed 27.8% and 16.7% of patients achieving prolonged clinical benefit at 4 and 6 months, respectively. The average duration of response for patients achieving clinical benefit for 4 months was 6.5 cycles. The prolonged benefit observed in a subset of patients is meaningful, given the aggressive nature of TNBC and low response rates to single-agent chemotherapies used in clinical practice. In a retrospective multicenter review of 111 patients with metastatic TNBC treated with standard-of-care chemotherapy, Kassam et al. found the median duration of therapy to be 9 weeks in the second-line setting and just 4 weeks in the third-line setting. Only 50% of patients went on to receive third-line therapy [4]. In a more recent analysis of the comparative effectiveness of eribulin, capecitabine, gemcitabine, and navelbine in metastatic previously treated TNBC, Dranitsaris et al. reported a median duration of therapy of 1.6–2 months for these agents [14]. There remains a critical unmet need for effective, targeted therapies to treat TNBC, and to achieve this will likely require patient selection strategies to identify a responding population for new therapies.

ENMD-2076 treatment resulted in mechanism-based toxicities, most commonly hypertension, fatigue, and diarrhea, consistent with prior clinical trials in solid tumor patients [10, 15]. Hematologic toxicity was not common in this study. The majority of patients (66%) experienced hypertension, and for 39% it was grade 3 or higher. This incidence and severity are similar to rates observed in prior studies of ENMD-2076 in patients with cancer and is likely related to VEGFR2 inhibition [10, 15]. In most cases, hypertension was easily controlled with antihypertensive agents, most commonly a dihydropyridine calcium channel blocker or an angiotensin-converting enzyme inhibitor. Close monitoring of blood pressure and careful management of hypertension should continue to be required in future studies of ENMD-2076.

Despite lowering the starting dose in this trial from that used in the phase II ovarian cancer trial, dose reduction was required in 20% of patients, and 12.2% of patients discontinued treatment owing to treatment-related toxicity [10]. These rates of toxicity and dose modification are similar to those for other approved multitarget tyrosine kinase inhibitors, including sorafenib [16]. Limited PK analysis performed in this study demonstrated a mean steady-state plasma concentration of ENMD-2076 at cycle 2, day 1 of 441 ± 394 ng/ml, which is similar to 356 ng/ml observed in patients with ovarian cancer [10]. The toxicity observed in this trial should be considered in the design of future studies of ENMD-2076, and the incorporation of standard toxicity management algorithms may assist in the management of hypertension.

This trial incorporated archival tumor tissue analysis for p53 mutation and protein expression, as well as serial tumor biopsies in a subset of patients. These correlative studies were designed to further delineate the mechanism of action of this multitarget drug using human tumor samples and to explore a potential relationship between deleterious p53 mutations and ENMD-2076 activity in TNBC. Although these studies are exploratory in nature owing to a limited sample size, analysis can be performed in the context of previously published preclinical studies [7, 8, 13].

As expected, ENMD-2076 was antiangiogenic, as evidenced by a decrease in blood vessel formation in tumor tissue following 2 weeks of treatment. ENMD-2076 is a potent inhibitor of the angiogenic kinases VEGFR2 (half-maximal inhibitory concentration [IC_50_], 58 nM), VEGFR3 (IC_50_, 16 nM), FGFR1/2 (IC_50_, 71 and 93 nM, respectively), and platelet-derived growth factor receptor-α (IC_50_, 56 nM) [7]. We previously demonstrated in vivo that administration of ENMD-2076 treatment results in a decrease in MVD, as well as vascular permeability and perfusion, as measured by dynamic contrast-enhanced MRI [17]. TNBC commonly overexpresses vascular endothelial growth factor, which acts to promote angiogenesis and early metastatic potential [18, 19]. Similar to other trials, this study did not demonstrate a correlation between a decrease in MVD and clinical benefit. This highlights the multifaceted mechanisms of cancer progression and that inhibiting angiogenesis alone is unlikely to be sufficient to lead to durable tumor response in TNBC.

A decrease in cellular proliferation that was also independent of response to therapy was observed and is likely a result of Aurora kinase inhibition. ENMD-2076 is more selective for Aur A than Aurora kinase B (IC_50_, 14 nM and 350 nM, respectively); however, inhibition of both is likely in tumor tissue, based on the drug exposure observed in this study. The early time point selected for the on-treatment biopsy may have resulted in capturing a decrease in cellular proliferation that occurred early and was lost quickly with tumor progression at the 2-month repeat imaging time point. As expected, induction of apoptosis was observed only in association with a favorable response to treatment, consistent with preclinical models [13]. ENMD-2076 treatment-emergent increases in p53 and p73 expression were observed, also consistent with preclinical data.

We observed p53 mutations in the majority of patients, as expected on the basis of the known genomic landscape of TNBC [20]. Deleterious mutations in p53 were more common in patients with clinical benefit for > 6 months; however, this was not statistically significant, owing to the small sample size. An increased sensitivity of p53-deficient models to Aurora kinase inhibitors, including AZD1152, AMG 900, and ENMD-2076, has been previously reported [7, 21, 22]. This is likely related to an impaired p53-dependent cell cycle checkpoint, which results in cells continuing to cycle through aberrant mitoses following drug exposure, ultimately resulting in cell death.

In this study, we observed a relatively high incidence of asymptomatic brain metastasis. Approximately 15% (10 of 65 patients consented) of patients in this trial had asymptomatic brain metastasis detected on the required screening brain MRI. Patients with TNBC are more likely than patients with hormone receptor-positive, HER2-negative breast cancer to develop brain metastasis. Approximately 50% of patients with metastatic TNBC will develop brain metastasis over the course of their disease [23–26]. These patients were not eligible for enrollment in this trial, owing to concerns regarding the risk of hemorrhagic events at the time of trial conception; however, data are now available to support the safety of antiangiogenics in asymptomatic brain metastasis [27]. Patients with stable, asymptomatic brain metastasis previously treated with radiation therapy could be considered for future trials of ENMD-2076. The propensity for asymptomatic brain metastasis should be considered in the design of future metastatic TNBC trials.

## Conclusions

ENMD-2076 has meaningful clinical benefit in a small subset of patients with previously treated metastatic TNBC. Future studies should continue to evaluate the relationship between p53 and response to treatment with a focus on the development of a patient selection strategy. Currently ongoing studies include a phase II clinical trial of ENMD-2076 in patients with metastatic previously treated TNBC in Asia, and biomarker development work is ongoing. This trial demonstrates the feasibility of performing serial tumor biopsies in multisite trials in metastatic TNBC and highlights the importance of these studies in confirming data generated in preclinical models. Combination therapies with immunotherapy and mammalian target of rapamycin pathway inhibitors are also under investigation based on ongoing preclinical work [8].
